# Alterations in androgen deprivation enhanced prostate-specific membrane antigen (PSMA) expression in prostate cancer cells as a target for diagnostics and therapy

**DOI:** 10.1186/s13550-015-0145-8

**Published:** 2015-11-17

**Authors:** B. Meller, F. Bremmer, C. O. Sahlmann, S. Hijazi, C. Bouter, L. Trojan, J. Meller, P. Thelen

**Affiliations:** Department of Nuclear Medicine, Georg-August-University Göttingen, Robert-Koch-Str. 40, 37075 Göttingen, Germany; Department of Pathology, Georg-August-University Göttingen, Göttingen, Germany; Department of Urology, Georg-August-University Göttingen, Göttingen, Germany

**Keywords:** Prostate-specific membrane antigen, ^68^Ga peptide, Prostate cancer, Abiraterone acetate, Androgen receptor, Androgen deprivation

## Abstract

**Background:**

Prostate-specific membrane antigen (PSMA) is a promising target for diagnostics and therapy of prostate carcinoma (PCa). Based on the hypothesis that PSMA expression can be modulated by variations in androgen deprivation therapy (ADT), we investigated the binding of a PSMA-directed radiopharmaceutical in vitro in order to get an insight of the interactions between altered premedication and PSMA expression before repetitive PSMA-directed PET/CT for therapy response and targeted therapy implementation.

**Methods:**

The human castration-resistant PCa cell line VCaP (CRPC) was treated with either 1 nmol/L testosterone (T) over 20 passages yielding the androgen-sensitive cell line (revCRPC) or with 5 μmol/L abiraterone acetate (AA) generating the abiraterone-tolerant subtype CRPC_AA_. In these cell lines, T and AA were varied by either supply or withdrawal of T and AA. PSMA expression of the three cell culture models was detected by Western blot and immunohistochemical staining. For quantitative measurement of tracer uptake, 0.3 nmol/L ^68^Ga-labelled PSMA-HBED-CC peptide (100–300 kBq/ml) was added to different treated parallel cultures (*n* = 9 each). Time-dependent uptake per 10^6^ cells of each culture was calculated and evaluated. PSMA mRNA expression was investigated by qPCR.

**Results:**

PSMA expression increased dependently on intensified ADT in all three basic cell lines. ^68^Ga-PSMA-HBED-CC uptake almost doubled during 3 h in all cell lines (*p* < 0.01). Compared to the basic cells, pre-incubation with abiraterone for 48 h resulted in a significant increased uptake in CRPC (*p* < 0.001). In revCRPC, 48-h AA pre-incubation resulted in an eightfold higher uptake after 3 h (*p* < 0.001). Additional withdrawal of external testosterone increased the uptake up to tenfold (*p* < 0.01). The increase of PSMA expression upon ADT and AA treatments was confirmed by qPCR and Western blot data. Furthermore, in CRPC_AA_, 48-h AA withdrawal increased the uptake up to fivefold (*p* < 0.01).

**Conclusions:**

The investigated three PCa cell culture subtypes represent a serial preclinical model of androgen deprivation therapy as a proxy for clinical situations with differing basal PSMA expression. The uptake of PSMA-binding tracers could be stimulated by therapeutic effective short-term variation in premedication in all stages of ADT response. These complex interactions have to be considered in the interpretation of diagnostic imaging using PSMA ligands as well as in the optimal timing of PSMA-based therapies.

**Electronic supplementary material:**

The online version of this article (doi:10.1186/s13550-015-0145-8) contains supplementary material, which is available to authorized users.

## Background

Prostate cancer (PCa) became the most frequent cancer amongst men in Europe with an incidence increasing rapidly over the past two decades [1, 2]. Localised PCa can be curatively treated by radical prostatectomy or various radiotherapies and/or androgen deprivation therapy in some cases [3]. However, castration-resistant PCa (CRPC) from de novo metastatic or relapsed disease remains incurable even after the advent of recently developed highly active agents such as abiraterone (Zytiga®, an inhibitor of steroidogenesis) and enzalutamide (Xtandi®, a second-generation androgen receptor antagonist). Moreover, very recently, the sequential use of individual potent agents confronted therapists with still insufficiently explained the cross-resistance phenomena [4]. Under such conditions, when de facto highly potent therapeutics are compromised by prior use of another potent agent, indicative biologic markers for the usefulness of a subsequent therapy are required. Recently, prostate-specific membrane antigen (PSMA) has been introduced as an indicator of androgen receptor signalling in PCa by PET imaging [5]. In this study, we introduce PSMA as potential therapeutic target after exhaustive androgen deprivation therapy with abiraterone acetate, an inhibitor of steroidogenesis.

PSMA with the biologic function of glutamate carboxipeptidase II (GCPII) is a membrane-bound zinc metalloenzyme and is expressed in various tissues. GCPII catalyses the reaction of n-acetylaspartylglutamate (NAAG) with water to result in n-acetylaspartate (NAA) and glutamate [6]. While the role of this enzyme in central and periphery nervous system seems to be well understood, its role in prostate and prostatic cancer cells as well as in neovascularity of other tumours is still under discussion. Glutamate as a potent nitrogen source in biosynthesis, and aspartate plays a central role in the synthesis of androgens. Other functions of this protein like its folate hydrolase activity may have an additional impact for tumour growth [7].

Since the expression of PSMA in the prostate is nearly 100-fold higher than in other tissues and prostate cancer exhibits a tenfold higher expression than the healthy prostate, PSMA has become a conspicuous target for nuclear medicine diagnostics and therapy very recently. Since the 1990s several radiopharmaceuticals directed against PSMA were established [8–12]. Today several groups use PSMA-based PET for the diagnostics of PCa with excellent sensitivity based on a very high contrast [13–17]. Consequently, the preferable imaging quality and clear visualisation resulted in questions about therapy response especially in patients with metastatic PCa during androgen deprivation therapy (ADT). During the last decades, choline analogues labelled with ^11^C and ^18^F were the ‘gold-standard’ in nuclear medicine PCa imaging [18, 19]. Furthermore, they were used for staging and therapy monitoring. Nevertheless, 2 years ago, Dost et al. reported data about the influence of ADT on choline PET/CT and commented on the lack of in vitro data on that issue [18].

To avoid repetition of that problem we focused on provision of basic investigations prior to ubiquitary use of PSMA-directed tracers. There are basic information of the influence ADT on PSMA expression in vivo [20]. For castration-resistant cancer cells, it has already been described that ADT increases PSMA expression [21]. Recently, hormone withdrawal combined with additional suppression of endogenous androgen synthesis and/or blockade of androgen receptors is one major strategy in PCa therapy [3, 22]. However, only a small portion of patients scheduled for PSMA-PET/CT suffered from castration-resistant PCa. These patients are usually investigated based on increasing PSA serum level prior or after therapy alterations. Whereas the impact of increasing standardized uptake value (SUV) and/or detection of additional lesions after therapy variation in these patients is unknown in that particular situation.

Various patients will receive their primary scan prior to prostatectomy in ADT-naïve situation. Follow-up with PSMA PET/CT will be performed based on very low and slightly increasing PSA serum levels, perhaps for planning of pelvic lymph node dissection. If ADT should be performed prior to PSMA PET/CT is as yet unclear.

Some patients will be scheduled for PSMA-based radionuclide therapy subsequent to ADT. It remains a central question if these patients will profit from ADT intermission.

Therefore, in vitro data for the whole spectra of potential clinical situations from hormone-naïve prostate cancer to androgen-deprivation-tolerant PCa should be available. For this aim, our group established a cell culture system, which reflects several aspects in PCa therapy re-establishing an androgen-sensitive subtype (revCRPC), the castration-resistant CRPC origin and the abiraterone-tolerant subtype. In this system, we focused on the time dependency of the uptake, the changes of PSMA expression as well as uptake of a PSMA-binding radiopharmaceutical after supply and withdrawal of testosterone and abiraterone acetate to avoid misinterpretations in vivo. Additionally, the clinical long-term objective is stimulation of PSMA expression by premedication sensitising the detection of primary and recurrent PCa and its metastases for diagnostics as well as for PSMA-directed therapy.

## Methods

### Radiolabeling of PSMA-HBED-CC

Elution of a ^68^Ge/^68^Ga-Generator (iThemba Labs, Cape Town, South Africa) was performed with 6 mL 1 M HCl, and the eluate was diluted with 6 mL H_2_O. ^68^Ga was retained on a cation exchange cartridge (Reagent and Hardware Kit SC-01, ABX, Radeberg, Germany) in a GRP Module (Scintomics, Lindach, Germany). *M* = 1200 (600–2000) MBq ^68^Ga was eluted from the resin with 1.5 mL 5 M NaCl. Radiolabeling of 5 μg (5.3 nmol) PSMA-HBED-CC in 3 mL 1.5 M HEPES (Kit components, ABX, Radeberg, Germany) was performed during 10 min at 95 °C in a reaction vessel for single use from the kit. Purification of the product was performed as already described by Eder et al. [23]. Radiochemical purity was determined by TLC and HPLC (Additional file 1). In contrast to the described methods, we used an Agilent 1200 Series HPLC equipped with a 250 × 4.6 mm Lichrosorb RP C_18_, 5 μm column (NORDANTEC, Bremerhaven, Germany) with the described mobile phases (A aqueous 0.1 % trifluoroacetic acid (TFA), B acetonitrile with 0.1 % TFA) and a linear gradient starting with 100 % A to 90 % B after 12 min and followed by 3 min equilibration with 100 % A, flow rate 1 mL/min.

### Cell culture subtypes

All cells were cultured in 25 cm^2^ culture flasks in 7-ml DMEM medium supplemented with 1 % l-glutamine (Lonza, Basel, Switzerland), 10 % FCS (PAA, GE Healthcare Bio-Sciences, Uppsala, Sweden) and 2 % sodium pyruvate (Gibco, Karlsruhe, Germany). Cell cultures were used below 40 passages after subtype development.

Castration-resistant prostate cancer (CRPC): the human cell line VCaP derived from a hormone-refractory bone metastasis was obtained from the American Type Culture Collection (ATCC; CRL-2876, Wesel, Germany). This cell line proliferates without androgen supply.

Revert castration-resistant prostate cancer (revCRPC): this cell line represents the pre-CRPC phenotype and was subcultured with 1 nmol/L testosterone (Sigma, St. Louis, MO, USA) over at least 20 passages resulting in a steady androgen-sensitive subtype for experiments within the subsequent 10 passages [24].

Abiraterone acetate tolerant castration-resistant prostate cancer (CRPC_AA_): CRPC_AA_ was obtained by sub-culturing CRPC with 5 μmol/L abiraterone acetate over at least 20 passages.

### Western blot analysis of the cell culture subtypes

Total protein lysates were prepared using RIPA buffer with protease inhibitors (Roche, Mannheim, Germany) and were quantified using the Bio-Rad DC Protein Assay (Bio-Rad, Hercules, California, USA). The samples were prepared for gel electrophoresis by mixing each with 4× Laemmli buffer (Bio-Rad). Each well of the gel was loaded with 15 μg protein. The gel was run for approximately 30 min at 250 V in the Mini-PROTEAN® Tetra System with the POWER PAC 300 (both Bio-Rad). The blotting step was carried out with the Bio-Rad Trans-Blot®Turbo™ RTA Transfer kit onto a nitrocellulose membrane. The membrane was blocked with 5 % milk powder in TBS buffer with 0.5 % Tween for 1 h at room temperature. For protein analysis, the primary antibody against PSMA (Dako M3620, monoclonal mouse, diluted 1/500, overnight at 4 °C) was used. The housekeeping gene α-tubulin (monoclonal mouse, diluted 1/5000) was labelled at room temperature for 1 h. The primary antibodies were detected with HRP-labelled anti-mouse secondary antibodies (Dako, dilution 1/1000). The signal development was made visible by applying a chemiluminescent substrate (ECL system by Amersham Bioscience, Freiburg, Germany).

### Immunohistochemistry—cell blocks

Immunohistochemical analyses were performed as described previously [25]. Adherent tumour cell lines were trypsinised from culture flasks and washed with PBS. Cells were spun down at 300*g* for 5 min. The pellet was fixated with 1 ml 4.5 % formalin for 1 min through resuspension. After another centrifugation, the formalin was removed, and the cell pellet was resuspended in 200 μl liquid 3 % agarose gel. The gel pellets were dried overnight at room temperature, then the hardened gel cones were removed from the cups and cut lengthwise. These gels cones were put into tissue capsules for the pre-treatment and following paraffin embedding. Paraffin blocks were cut at 3 μm for immunohistochemistry. Sections were stained using a Dako autostainer with the Dako EnVision™ FLEX+ detection system (Dako, Glostrup, Denmark). The system detects primary mouse and rabbit antibodies, and the reaction was visualised by EnVision™ FLEX DAB+ Chromogen. Using EnVision™ FLEX+ Mouse (LINKER) or EnVision™ FLEX+ Rabbit (LINKER) (Code K8019), signal amplification of primary antibodies can be achieved. Deparaffinisation, rehydration and heat-induced epitope-retrieval (HIER) was carried out in one step with the 3-in-1 procedure buffer (Dako, Glostrup, Denmark, Target Retrieval Solution), pH 9 high ((10×) (3-in-1) Code S2375)) at 97 °C using a PT Link, Pre-Treatment Module 6 (Dako). Tissue samples were analysed by light microscopy after 8 min counterstaining with Meyer’s haematoxylin (Dako). As primary antibodies, we used anti-PSMA (Dako M3620, monoclonal mouse antibody, diluted 1:50, 30 min).

### Uptake measurements and cell count

All uptake experiments were carried out in three separate series resulting in *n* = 9 culture flasks for each cell line, variation in medication and measurement time. To assess the capacity of cells for the labelled compounds in one series, 0.3 nmol/L ^68^Ga-labelled peptide (100-300 kBq/ml) was simultaneously applied to three parallel cells cultures for each interval (1–3 h) and each cell line. Since activity yield from generator, yield of synthesis as well as delay between synthesis and cell culture experiments differed between the experimental series, peptide amount kept constant between the separate series. Before measuring the tracer uptake, culture medium was discarded, and the cells were washed twice with phosphate buffered saline (PBS) buffer pH 7.4. The cells were dissociated by accutase/EDTA (PAA, GE Healthcare Bio-Sciences, Uppsala, Sweden) and centrifuged. Activity of cell pellet and collected supernatants was measured. Data were half-life-corrected, and percentage uptake of applied activity was calculated. Since differences in treatment resulted in altered proliferation, the calculated uptake was correlated to the determined cell count in each particular culture flask and normalised to 10^6^ cells as already described [26, 27].

### Variations of abiraterone acetate and testosterone treatment

CRPC and revCRPC cells were incubated over 48 h with 5 μmol/L Abiraterone acetate (Janssen-Cilag, Neuss Germany) prior to activity supply. In parallel revCRPC cultures abiraterone acetate (AA)-induced androgen deprivation was aggravated by testosterone withdrawal. In CRPC_AA_ cells, AA was eliminated from culture medium and 1 nmol/L testosterone was supplied.

### Analysis of mRNA expression

mRNA expression of 10^6^ cells per sample were analysed by qRT-PCR. Total cellular RNA from cultured cells was extracted with the Quick-RNA™MiniPrep (Zymo Research, Freiburg, Germany). Total RNA integrity and quantity were assessed on an Experion™ Automated Electrophoresis System with analysis kits consisting of microfluidic chips and reagents (Bio-Rad, Munich, Germany). Reverse transcription of 500 ng total cellular (tc) RNA was performed with random hexamer primers and an Omniscript RT Kit (QIAGEN, Hilden, Germany). Expression analyses were processed on a CFX96™ real-time detection system (Bio-Rad, Munich, Germany) with SsoAdvanced Universal SYBR Green supermix. The 20 μl reaction mix from the kit was supplemented with 2 μl cDNA, 0.6 μM gene-specific primers (IBA, Goettingen, Germany). Primers were designed by Primer 3 [http://primer3.ut.ee] and assessed for PCR efficiency.

Time courses of gene expression (Fig. 3) were calculated as percent expression using the analysis of relative gene expression by real-time quantitative PCR and the 2(-ΔΔC_(T)_) method [28]. The *housekeeping gene* for calculation of relative expression was acidic ribosomal protein (ARP) and for proof of principle of this calculation another *housekeeping gene* ribosomal protein L13a (RPL) was used.

#### Internalisation

To elucidate if the three different subtypes or modification of medication resulted in different internalisation rates, a protocol for the detection of endocytosis in adherent cell lines was modified [29]. CRPC, CRPC_AA_, rev-CRPC as well as rev-CRPC under ADT (with abiraterone acetate and testosterone withdrawal) were investigated. Therefore, three cultures flasks (25 cm^2^) for each subtype and modification were seeded with 3 × 10^5^ cells and allowed to attach overnight in their specific mediums as described above. All cultures were grown for an additional 48 h whereby ADT was performed for three flasks as already described above. Cultures were incubated with 0.3 nmol/L ^68^Ga-HBED-CC for 5 h. In contrast to the mentioned protocol, adherent cells were harvested with trypsin/EDTA (PAA). Total activity was determined in culture medium, in the fraction of stripped radiolabel from cell surface proteins and the harvested cells. Thereafter, cells were spun down and total cell count was determined. Results were calculated as percentage of fraction activity normalised to 10^6^ cells of applied activity after half-life correction.

### Statistical analysis

Statistical analyses (Statistical Analysis Software: SPSS Statistics 22.0 for Windows) were performed using non-parametric tests (Wilcoxon, Mann-Whitney). Means (*m*) and standard deviations (SD) were calculated. Differences were considered significant when *p* < 0.05.

## Results

The radiolabelling of PSMA-HBED-CC with ^68^Ga was performed in high radiochemical purity (*m* = 98.7 %, range 95.7–99.9 %) and yield (*m* = 379 MBq, range 290–468 MBq) with specific activities of *m* = 71.5 GBq/mmol (range 54.7–88.3 GBq/mmol).

**Fig. 1:**
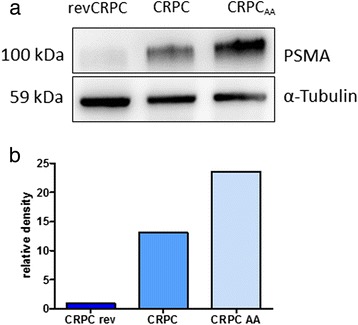
Western blot total protein extraction of the three cell lines subtypes with different levels of androgen deprivation sensitivity (revCRPC, CRPC, CRPC_AA_). Displayed is total PSMA protein at 100 kDa and the housekeeping gene α-tubulin (**a**). Relative density was determined by a chemiluminescent substrate (**b**)

PSMA protein expression was visualised by Western blot in all three different cell lines at 100 kDa (Fig. 1a). The lowest PSMA protein expression was observed in the androgen-sensitive cells by chemiluminescent detection. The highest expression was detected in the abiraterone-tolerant variant (Fig. 1b). The housekeeping gene α-tubulin tended slightly to decrease in the same order.

**Fig. 2:**
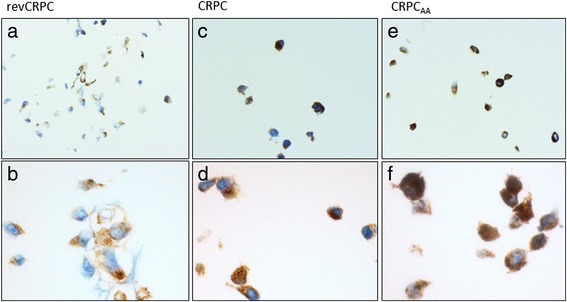
Immunohistological staining of PSMA in the three investigated cell lines subtypes. Cytoplasmic PSMA (*brown signal*) in revCRPC (**a** ×20, **b** ×60), CRPC (**c** ×20, **d** ×60) and CRPCAA (**e** ×20, **f** ×60)

Different PSMA protein expressions could be confirmed by immunohistochemical staining of paraffin embedded cells (Fig. 2). In revCRPC, cells were only partially stained by PSMA antibodies (Fig. 2a/b). With increasing castration, resistance staining increased within cell lines (CRPC (Fig. 2c/d ) <CRPC_AA_ (Fig. 2e/f )).

**Fig. 3:**
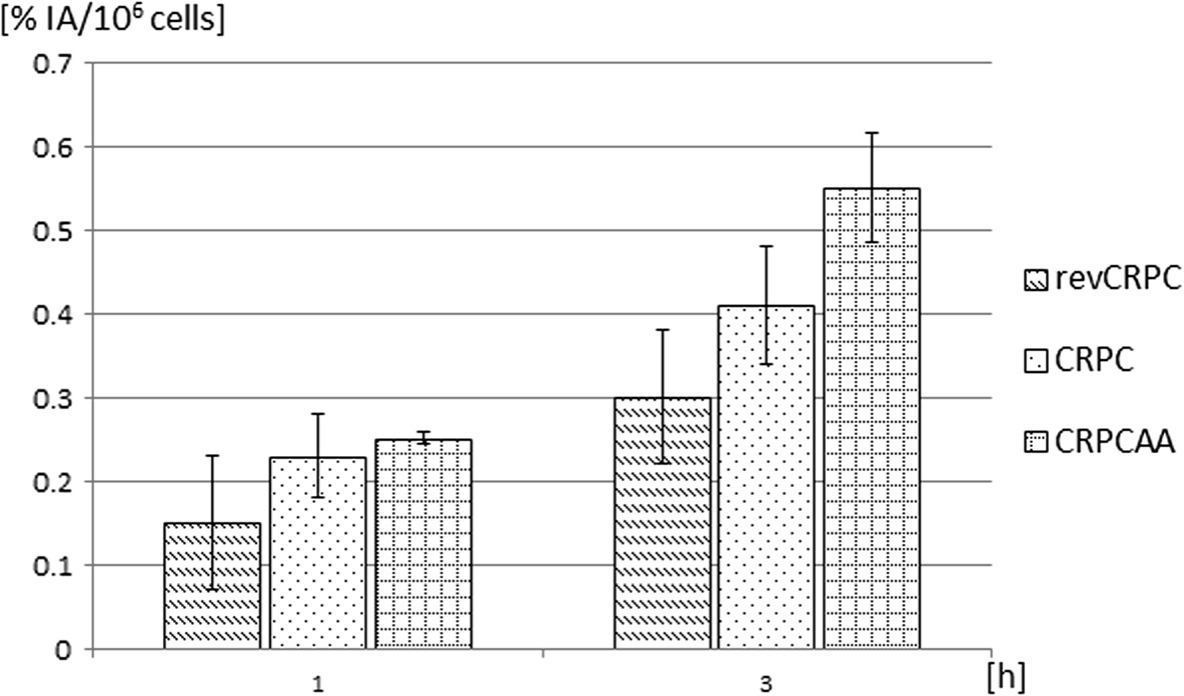
Time-dependent uptake of ^68^Ga-PSMA-HBED-CC in androgen-sensitive (revCRPC), castration-resistant prostate cancer cells (CRPC) and abiraterone-insensitive cells (CRPC_AA_). Displayed is the percentage uptake of applied radioactivity per 10^6^ cells (mean ± SD; *n* = 9 cultures)

With the radiolabelled peptide, the ascending order of PSMA expression according to increasing androgen deprivation tolerance could be affirmed. Androgen-sensitive cells exhibited lower uptake than the castration-resistant cells. The highest uptake was determined for abiraterone-tolerant cells. The observed differences between PSMA uptakes were significant for all the different experimental groups after 3 h (*p* < 0.05). The half-life-corrected uptake of untreated cell lines increased significantly over time (*p* < 0.05). The uptake of the labelled compound in the three subtypes was on a relatively low level after 1 h but doubled after 3 h of incubation (Fig. 3).

**Fig. 4:**
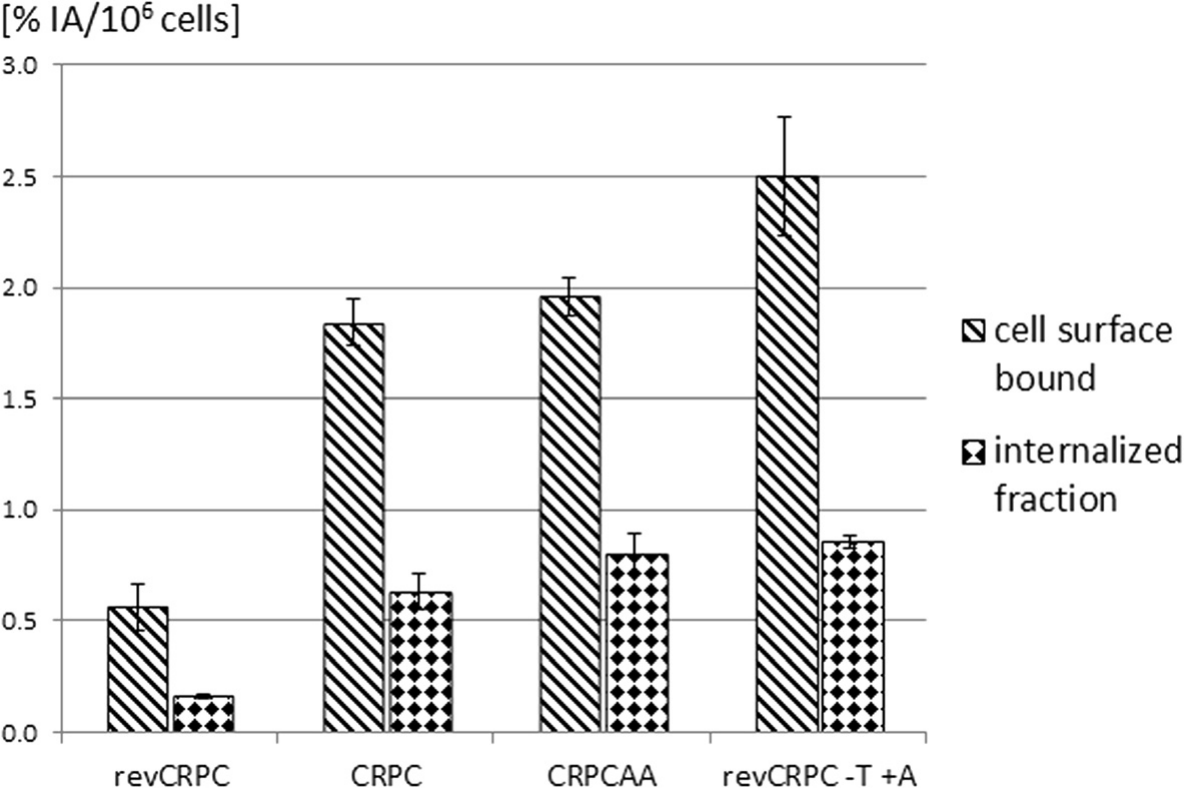
Membrane binding and internalisation of ^68^Ga-PSMA-HBED-CC after 5 h of incubation in androgen-sensitive (revCRPC), castration-resistant prostate cancer cells (CRPC) and abiraterone-insensitive cells (CRPC_AA_) as well as in revCRPC after 48 h ADT with testosterone (T) withdrawal and 5 μM abiraterone acetate (AA). Values are expressed as percentage of applied radioactivity bound to 10^6^ cells. Mean ± SD (*n* = 3 cultures)

Internalisation experiments revealed a further increase of total cell bound activity after 5 h compared to activity after 3 h. Percentage of internalised peptide was about 30 % of membrane-bound activity in the investigated three subtypes (Fig. 4).

### Variation in androgen deprivation

**Fig. 5:**
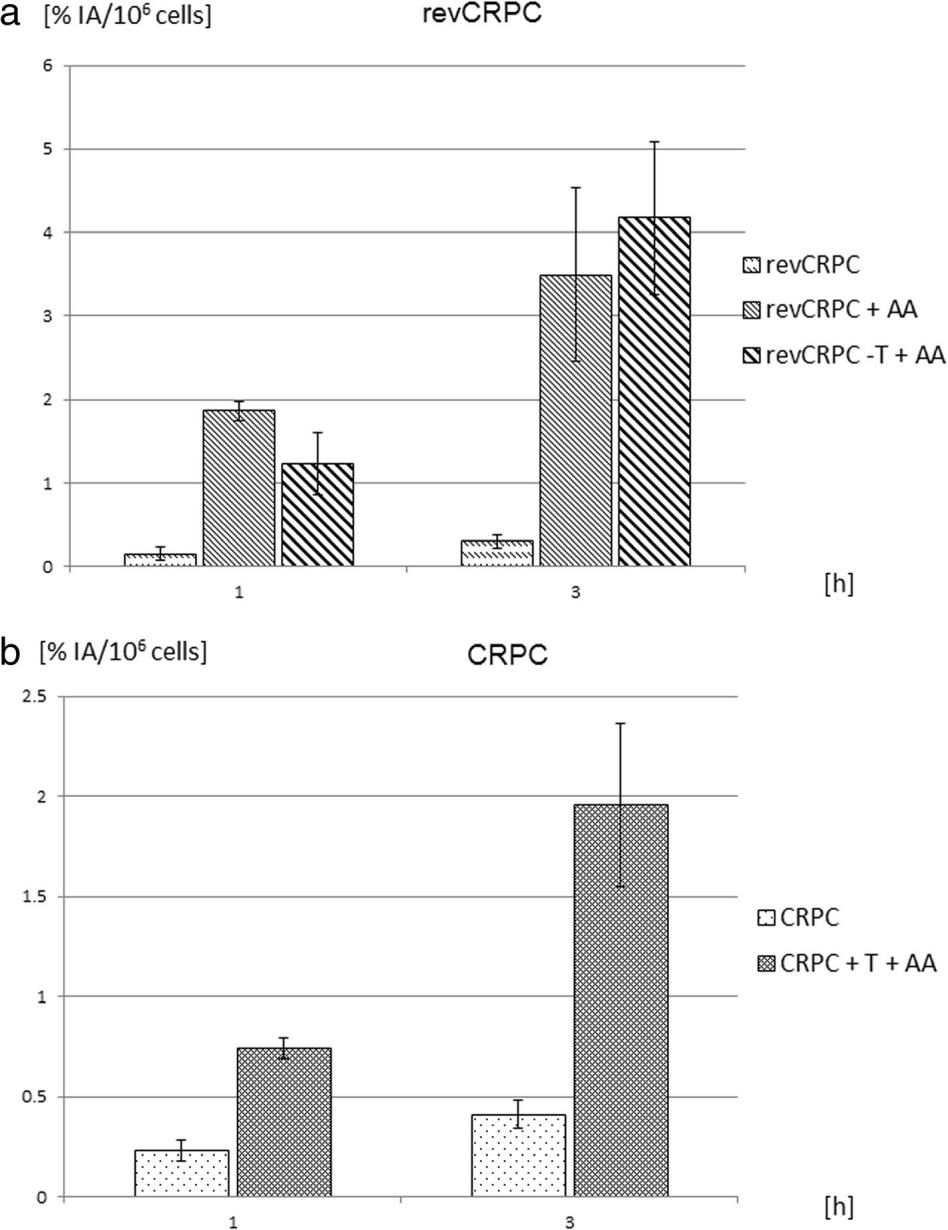
Time-dependent binding of ^68^Ga-PSMA-HBED-CC in (**a**) castration-resistant prostate cancer (CRPC) cells with and without coincubation with abiraterone acetate (AA, 5 μmol/L) and testosterone (T, 1 nmol/L) and (**b**) in androgen-sensitive revCRPC cells with and without AA-treatment (5 μmol/L) or additional withdrawal of testosterone (−T) from the medium. Displayed is the percentage uptake of applied radioactivity per 10^6^ cells (mean ± SD; *n* = 9 cultures)

Compared to control CRPC cells, treatment with 5 μmol/L abiraterone acetate (AA) over 48 h resulted in a significant increased uptake of ^68^Ga-PSMA-HBED-CC (*p* < 0.001) and decreased proliferation about 40 %. The uptake after 3 h increased more than fivefold (Fig. 5a).

In androgen-sensitive revCRPC cells, androgen deprivation treatment with 5 μmol/L AA resulted in an eightfold higher uptake after 3 h. Further withdrawal of testosterone from the medium (+AA −T) increased the uptake up to tenfold (*p* < 0.001, Fig. 5b). In internalisation experiments, differences induced by ADT were evident. In revCRPC, ADT induced an increase of membrane bound and internalised ^68^Ga-PSMA-HBED-CC (Fig. 4) whereby proliferation was significantly inhibited. The growth was <50 % in ADT-treated revCRPC compared to the revCRPC in the same assay. The activities in the different fractions of ADT-treated revCRPC cells were comparable to observed levels in CRPC and CRPC_AA_.

**Fig. 6:**
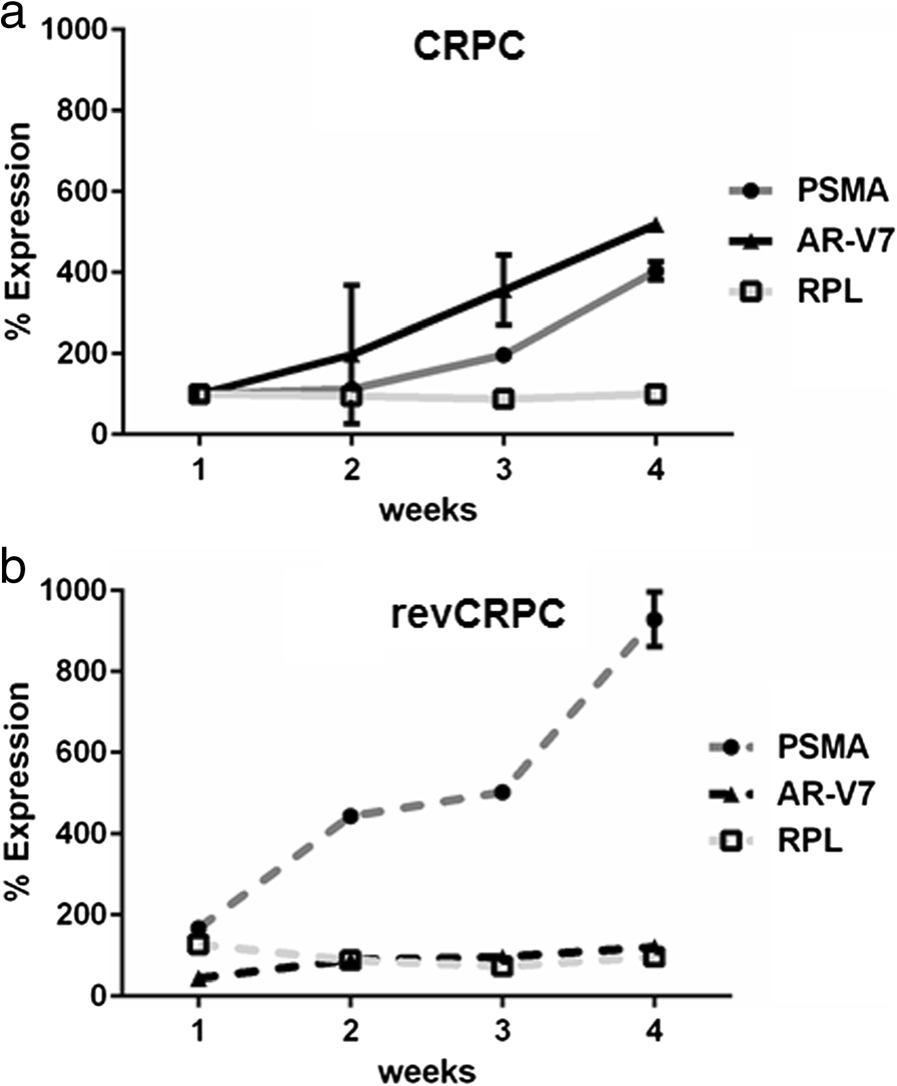
Time-dependent changes of PSMA mRNA expression, androgen receptor splice variant 7 (AR-V7) and ribosomal protein L13a (RPL) *housekeeping gene* in (**a**) CRPC cells under abiraterone acetate (AA) treatment and (**b**) revCRPC cells under AA treatment and additional androgen withdrawal from the medium

The influence of AA treatments on PSMA expression was confirmed by PSMA mRNA quantitation in CRPC cells. On the mRNA level, PSMA expression increased by more than 300 % within 4 weeks treatment with AA (Fig. 6a). In androgen-sensitive revCRPC cells, the AA treatment combined with androgen withdrawal resulted in an almost tenfold higher PSMA expression. RPL housekeeping gene expression was not altered under these treatments in both cell lines while androgen receptor splice variant 7 (AR-V7) also increased markedly in CRPC cells by AA treatment, whereas in androgen-sensitive revCRPC cells androgen deprivation was best indicated by PSMA expression (Fig. 6b).

**Fig. 7:**
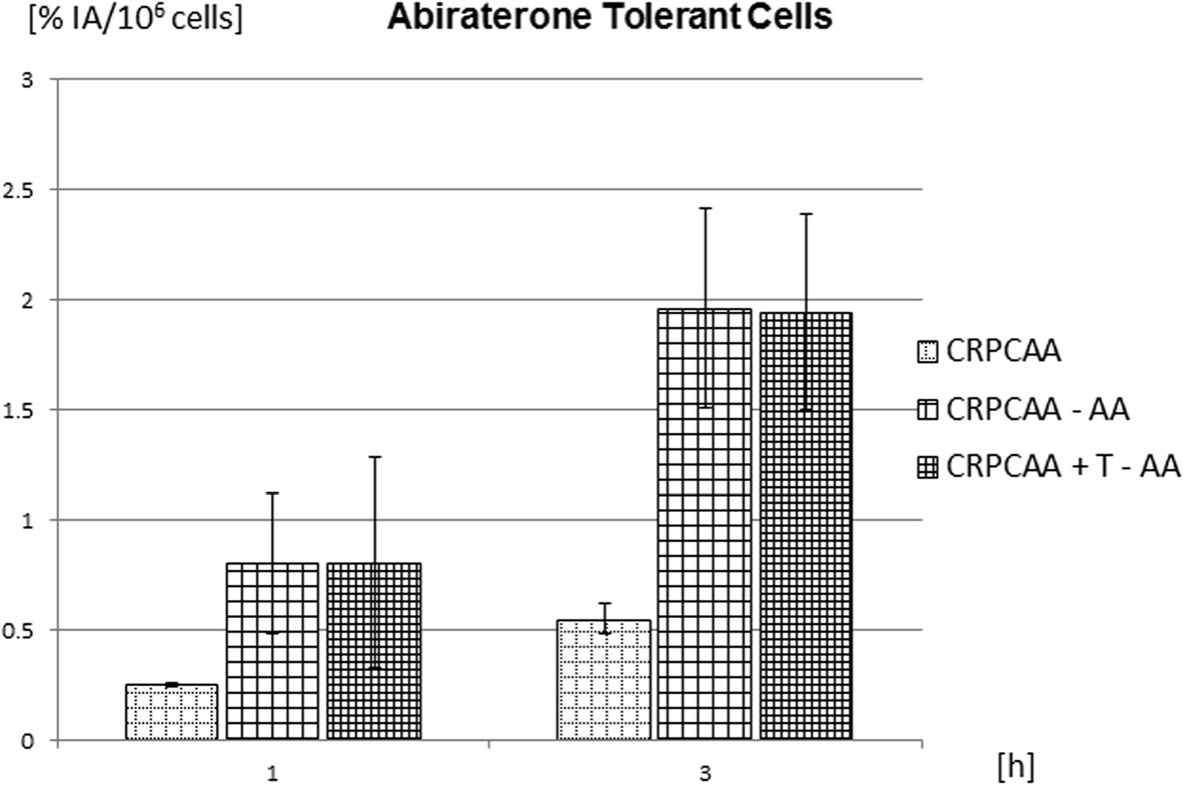
Time-dependent changes of ^68^Ga-PSMA-HBED-CC uptake 1 and 3 h after application in abiraterone-tolerant cells 48 h (CRPC_AA_) after abiraterone acetate (AA) withdrawal with or without additional application of 1 nmol/L testosterone (T). Displayed is the percentage uptake of applied radioactivity per 10^6^ cells (mean ± SD; *n* = 9 cultures)

Prolonged AA treatments of CRPC cells up to 7 months yielded another CRPC cell variant no longer susceptible to continuous AA treatment. This cell variant was termed CRPC_AA_. In contrast to revCRPC and CRPC whose androgen deprivation caused an increase of ^68^Ga-PSMA-HBED-CC-uptake (Fig. 5), CRPC_AA_ revealed an elevated uptake of the peptide upon cessation of androgen deprivation and androgen supplementation (Fig. 7). In this cell line, peptide uptake was significantly increased up to fivefold (*p* < 0.01) after 3 h of application, when AA had been withdrawn from cell culture media (−AA). Proliferation decreased in this cell line by about 25 % after AA withdrawal. This level of uptake remained constant when 1 nmol/L testosterone had been added to AA withdrawal medium (−AA + T).

## Discussion

PSMA is a novel and promising target for diagnostics and therapy of metastatic prostate cancer, especially when the androgen receptor is the persistent therapeutic target and pending cross resistances might occur in subsequent androgen-directed therapies [30]. Several groups focused on the relations between PSMA expression and other membrane antigens. Evans et al. found an androgen receptor (AR) dependency of PSMA expression. AR-negative PCa cell lines consequently lack PSMA expression [5]. They demonstrated a downregulation of PSMA mRNA expression in AR-positive cell lines after incubation with 10 nmol/L testosterone for 72 h. In our in vitro model, the basal 1 h uptake of PSMA-binding peptide differed between castration-resistant cells and the androgen-sensitive cell line. This is consistent with the notion of our cell model representing androgen signalling in revCRPC with low androgen receptor expression in the present of androgens (1 nmol/L testosterone) and CRPC with overexpressed androgen receptor in the absence of androgens [24, 31]. Our cell culture system represents different stages of castration resistance (including the androgen-sensitive and the AA-tolerant subtype) without the bias of common mutations in androgen receptor of most other PCa cell cultures.

Additionally, we were able to substantiate increasing PSMA expression by increasing androgen deprivation on the protein level. Differences between uptake measurements and Western blot visualisation by chemiluminescent staining could readily be explained by the differences in methods and substrates. While peptide uptake represents the sum of membrane-bound and internalised activity at varying kinetic speeds of both processes in growing cell cultures, Western blot and immunohistochemistry was performed with isolated proteins or fixed cells using PSMA antibodies.

Eder et al. described the internalisation of ^68^Ga-PSMA-HBED-CC [23, 32]. We could confirm these results and the ratio between the membrane-bound and internalised fraction of the tracer. In addition, we found an increasing uptake over time. Compared to the 1-h value, the half-life-corrected value 3 h after peptide administration was almost doubled. These findings were observed in all three cell line subtypes. This might be an important finding for in vivo applications, as we recently could demonstrate a higher ^68^Ga-PSMA-HBED-CC uptake 3 h after injection in metastatic and primary PCa, too [33]. An increase over time seems to be a criterion for differentiation between benign and malignant lesions. This might be meaningful for differentiation of very small lesions near by the detection limits in PET/CT and also for PSMA-directed therapy. During the last decades, several new potent therapeutic options for prostate carcinoma have been developed. This resulted in a decrease of prostate-carcinoma-related death despite an increasing incidence of the disease [2, 4]. The development of different castration regimen and the pharmaceutical blockade of the androgen receptor has been optimised and reached clinical application. Enzalutamide (Xtandi®) is a second-generation antiandrogen competing with ligand binding to the androgen receptor, and abiraterone acetate (Zytiga®) is an inhibitor of intratumoral steroidogenesis. In contrast, LHRH agonists inhibit androgen synthesis only in the testes but not in the adrenal and prostate cancer cells.

The first data of an increasing PSMA expression and androgen receptor activation by abiraterone acetate and enzalutamide were presented in a preclinical study by flow cytometry in CRPC [21]. Our data confirm this increase monitored on the mRNA level substantiating these results on the protein level. Furthermore, we demonstrated an increasing uptake of PSMA-binding tracer immediately after intensified androgen deprivation not only in CRPC but also in androgen-sensitive cell lines. In our internalisation experiments, the tracer binding properties of 48-h ADT-treated androgen-sensitive cells were comparable to those of ADT-tolerant cells, albeit tracer binding was concomitant with proliferation decrease. The intracellular blocking of androgen synthesis seems to increase not only the expression of the androgen receptor but also of PSMA. This was confirmed by our uptake measurements for the early phase and for long-term application over several weeks. Further investigations demonstrated the cross-talk between androgen receptor and phosphoinositide 3-kinase (PI3K) signalling pathway and enhanced AR signalling after inhibition of PI3K pathway [21]. Another investigation observed the regulation of PI3K pathway by PSMA knockdown [34]. Therefore, it can be hypothesised that the PI3K pathway is a link between PSMA and AR. Notwithstanding mechanistic details, the main prospect of this study is the positioning of PSMA as a potential therapeutic target clearly marked in PET under pending therapy failure when alternative subsequent therapy is inappropriate due to cross resistances.

In our experiments with AA-insensitive CRPC_AA_ cells, an increased peptide uptake could also be induced by AA withdrawal for 48 h. AA withdrawal inhibited proliferation in these cells considerably. This is in coincidence with clinical findings in the literature. Recently, a case of biochemical and metabolic response to AA withdrawal with decreasing tumour size and choline uptake after AA rechallenge for 3 months has been documented by ^18^F-choline PET/CT [35]. This could be explained by a decreased need of membrane biosynthesis in a shrinking tumour. In contrast, other groups reported that PSMA and the prostate-specific antigen (PSA) are inversely correlated under androgen deprivation [5, 36].

It is well known that ADT decreased PCa growth in androgen-sensitive tumours [3, 37]. In a castration-resistant situation, ADT cessation seems to decrease tumour proliferation, too [35]. Therefore, increasing PSMA expression might be a sign for tumour response after variation in androgen deprivation in general. Our preliminary study holds an explanation for this phenomenon in the overexpression of CYP17A1 and androgen receptor in CRPC_AA_ cells and normalised expression after AA withdrawal and supplementation with low concentrations of testosterone [38]. The underlying molecular mechanisms of these early effects have to be elucidated in further investigations.

## Conclusions

The investigated three PCa cell culture subtypes represent a serial preclinical model of androgen deprivation therapy as a proxy for clinical situations with differing basal PSMA expression. The uptake of PSMA-binding tracers could be stimulated by therapeutic effective short-term variation in premedication in all stages of ADT response. These complex interactions have to be considered in the interpretation of diagnostic imaging using PSMA ligands as well as in the optimal timing of PSMA-based therapies.

## Ethical approval

This article does not contain any studies with human participants or animals performed by any of the authors.

## Additional file

Additional file 1:
^**68**^ **Ga-HBED-CC Radio-HPLC.** Quality control of a regular Ga-HBED-CC synthesis. Radiochemical purity was >99.5 % and retention time of the product was 7.65 min. (PDF 26 kb)
