# Supplementary figures and images for: Alterations in androgen deprivation enhanced prostate-specific membrane antigen (PSMA) expression in prostate cancer cells as a target for diagnostics and therapy
Source: EJNMMI Res. 2015 Nov 17;5:66. doi: 10.1186/s13550-015-0145-8 (PMC4648835; doi:10.1186/s13550-015-0145-8)

# $^{68}\text{Ga}$ -HBED-CC Radio-HPLC

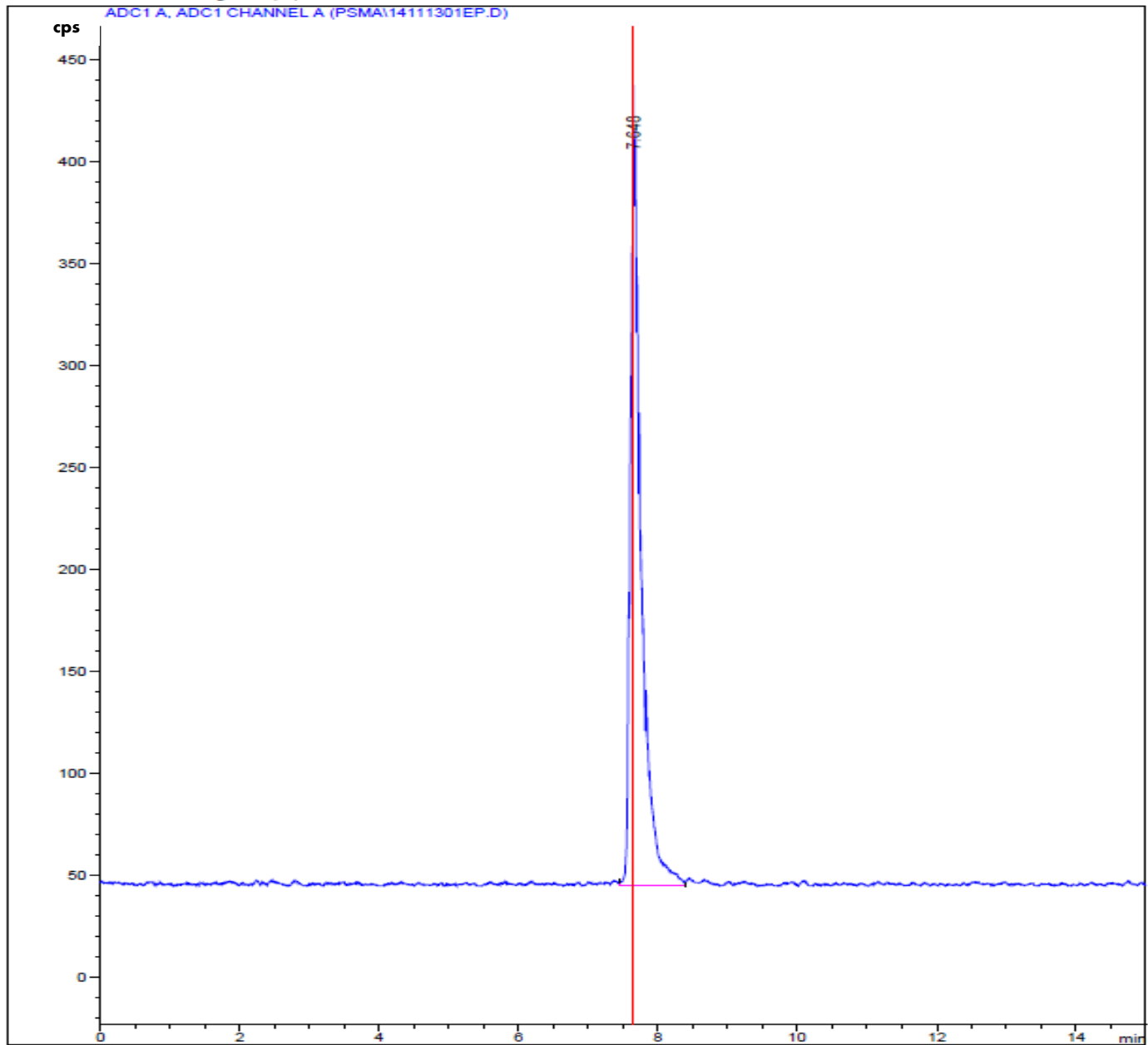

Supplement: Additional file 1: — 68 Ga-HBED-CC Radio-HPLC. Quality control of a regular Ga-HBED-CC synthesis. Radiochemical purity was >99.5 % and retention time of the product was 7.65 min. (PDF 26 kb) [file 13550_2015_145_MOESM1_ESM.pdf]
